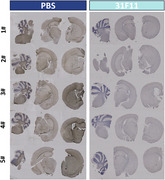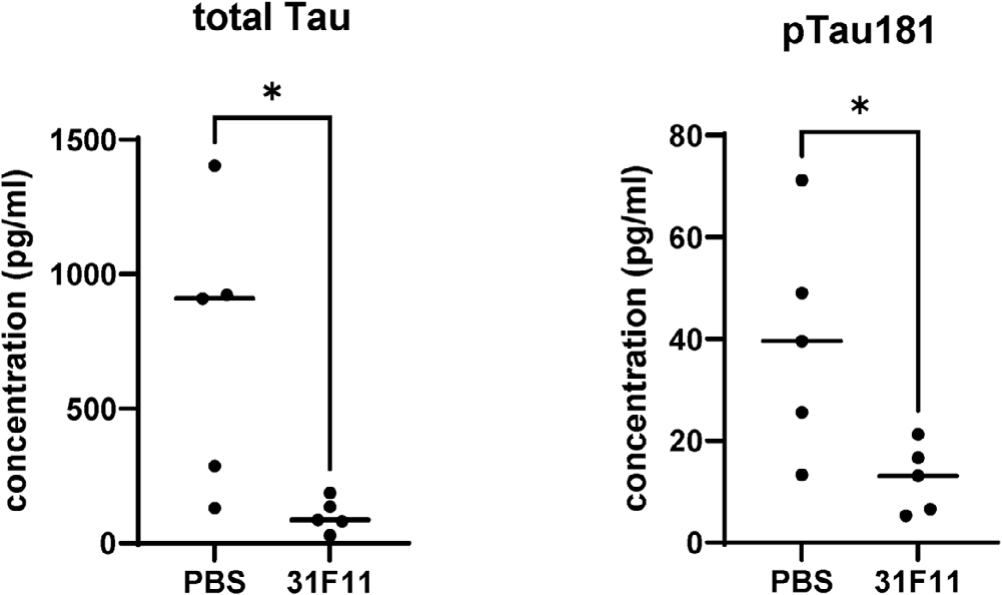# Therapeutic activities of anti‐human SIGLEC10 mAb in Multiple Transgenic Mouse AD Models

**DOI:** 10.1002/alz70859_101818

**Published:** 2025-12-25

**Authors:** Peng Wang, Timothy Esworthy, Ido Weiss, Pan Zheng, Yang Liu

**Affiliations:** ^1^ OncoC4, Inc., Rockville, MD USA

## Abstract

**Background:**

Microglial dysfunction is associated with Alzheimer’s disease (AD). However, no clinical benefit has been achieved by targeting microglia in AD patients. SIGLEC10 is exclusively expressed in the microglia in the brain. Our recent data suggest a critical role for SIGLEC10 in AD pathogenesis in the mice expressing human SIGLEC10. Here we investigated therapeutic activity of ONC‐841, an anti‐SIGLEC10 monoclonal antibody, in two AD models.

**Method:**

Two AD mouse models (ARTE and JNPL3) were crossed with human SIGLEC transgenic mice to create SIGLEC10 expressing AD models. To test whether ONC‐841 can pass blood brain barrier, ONC‐841 were injected intravenously, and the occupancy of microglial SIGLEC10 was tested by both flow cytometry and immunohistochemistry. To avoid xenoreactivity, a mouse IgG clone of ONC‐841(31F11) was produced and used to evaluate therapeutic activities. Accumulation of both pTau (AT8) and amyloid plaques (6E10) in the brain were examined by immunohistochemistry. Plasma pTau181 and total Tau were evaluated as biomarkers. The mechanism of action (MOA) was investigated through single nuclear RNA sequencing and in vitro phagocytosis.

**Result:**

Twenty weeks old ARTE10/Sg10 mice were intravenously treated with 31F11 or vehicle over 6.5 weeks. The brain sections from treated mice showed dramatic clearance of amyloid‐β plaque in comparison to controls (Figure 1). 31F11 also caused significant reduction of plasma pTau 181 and total Tau in JNPL3/Sg10 mice (Figure 2). In vitro phagocytosis assay revealed that ONC‐841 promote phagocytosis of both Ab and Tau aggregates by IPS‐derived human microglia and mouse primary microglia. Consistent with these data, morphological analysis revealed that 31F11 attenuated AD‐associated microglia abnormalities. Moreover, single nuclear RNAseq analysis showed that anti‐SIGLEC10 treatment rejuvenated phagocytic and migratory activities of microglia. Taken together, our data suggest that ONC‐841 protect against AD pathogenesis in the mice by rejuvenating microglia functions.

**Conclusion:**

In two AD mouse models, anti‐SIGLEC10 cleared the amyloid plaques or reduced the plasma p‐Tau biomarkers. The clinical stage anti‐SIGLEC10 antibody, ONC‐841, promotes phagocytosis of protein aggregates. ONC‐841 protects against AD pathogenic process in the mice by rejuvenating microglia functions. These studies provided the scientific foundation for further clinical development with ONC‐841 as potential microglia targeting immunotherapy agent for AD.